# ^18^FDG, [^18^F]FLT, [^18^F]FAZA, and ^11^C-Methionine Are Suitable Tracers for the Diagnosis and *In Vivo* Follow-Up of the Efficacy of Chemotherapy by miniPET in Both Multidrug Resistant and Sensitive Human Gynecologic Tumor Xenografts

**DOI:** 10.1155/2014/787365

**Published:** 2014-09-18

**Authors:** György Trencsényi, Teréz Márián, Imre Lajtos, Zoltán Krasznai, László Balkay, Miklós Emri, Pál Mikecz, Katalin Goda, Gábor Szalóki, István Juhász, Enikő Németh, Tünde Miklovicz, Gábor Szabó, Zoárd T. Krasznai

**Affiliations:** ^1^Department of Nuclear Medicine, University of Debrecen, P.O. Box 63, Nagyerdei Körút 98, Debrecen 4012, Hungary; ^2^Department of Biophysics and Cell Biology, University of Debrecen, Nagyerdei Körút 98, Debrecen 4012, Hungary; ^3^Department of Dermatology, University of Debrecen, Nagyerdei Körút 98, Debrecen 4012, Hungary; ^4^Department of Surgery and Operative Techniques, Faculty of Dentistry, University of Debrecen, Nagyerdei Körút 98, Debrecen 4012, Hungary; ^5^Department of Obstetrics and Gynecology, University of Debrecen, Nagyerdei Körút 98, Debrecen 4012, Hungary

## Abstract

Expression of multidrug pumps including P-glycoprotein (MDR1, ABCB1) in the plasma membrane of tumor cells often results in decreased intracellular accumulation of anticancer drugs causing serious impediment to successful chemotherapy. It has been shown earlier that combined treatment with UIC2 anti-Pgp monoclonal antibody (mAb) and cyclosporine A (CSA) is an effective way of blocking Pgp function. In the present work we investigated the suitability of four PET tumor diagnostic radiotracers including 2-[^18^F]fluoro-2-deoxy-D-glucose (^18^FDG), ^11^C-methionine, 3′-deoxy-3′-[^18^F]fluorothymidine (^18^F-FLT), and [^18^F]fluoroazomycin-arabinofuranoside (^18^FAZA) for *in vivo* follow-up of the efficacy of chemotherapy in both Pgp positive (Pgp^+^) and negative (Pgp^−^) human tumor xenograft pairs raised in CB-17 SCID mice. Pgp^+^ and Pgp^−^ A2780AD/A2780 human ovarian carcinoma and KB-V1/KB-3-1 human epidermoid adenocarcinoma tumor xenografts were used to study the effect of the treatment with an anticancer drug doxorubicin combined with UIC2 and CSA. The combined treatment resulted in a significant decrease of both the tumor size and the accumulation of the tumor diagnostic tracers in the Pgp^+^ tumors. Our results demonstrate that ^18^FDG, ^18^F-FLT, ^18^FAZA, and ^11^C-methionine are suitable PET tracers for the diagnosis and *in vivo* follow-up of the efficacy of tumor chemotherapy in both Pgp^+^ and Pgp^−^ human tumor xenografts by miniPET.

## 1. Introduction

The miniPET technique is a well-established noninvasive method to detect tumors and follow up the effect of therapy of tumors, using PET tumor diagnostic tracers. The PET tumor diagnostic tracers, like ^11^C-metionine, 2-[^18^F]fluoro-2-deoxy-D-glucose (^18^FDG), and (3′-deoxy-3′-[^18^F]fluorothymidine) (^18^F-FLT), are good candidates to measure the effect of the therapy. ^18^FDG, an analog of glucose, the most commonly used PET radiotracer in clinical oncology, allows visualization of the changes in the glucose metabolic rate in tumors [1–3]. ^18^FDG undergoes phosphorylation by hexokinase but can not pass through the rest of glycolysis, and it remains trapped in the cell. Increased cell proliferation is one of the main features of cancer cells. ^18^F-FLT is used as a PET tracer for visualization of cell proliferation. The trapping of ^18^F-FLT was demonstrated with the uptake of thymidine analogue after phosphorylation by thymidine kinase 1 (TK1) in the S phase of cell cycle [4, 5]. Another tumor diagnostic PET tracer is the ^11^C labelled methionine applied for the follow-up of the amino acid transport and metabolism in the tumor [6]. Radiolabeled nitroimidazole derivatives (e.g., [^18^F]fluoroazomycinarabinofuranoside (^18^F-FAZA) and [^18^F]fluoromisonidazole (^18^F-MISO)) validated markers to detect hypoxia in cancer cells. Nitroimidazoles labeled with ^18^F isotope can undergo an oxygen-reversible single-electron reduction under hypoxic conditions, forming reactive oxygen radicals that subsequently bind covalently to macromolecular cellular components and are trapped in the intracellular space; consequently they are suitable tracers for* in vivo* detection of hypoxia in tumors. Hypoxia in tumor seems to be an important prognostic factor of chemotherapy response [7].

Multidrug resistance (MDR) seems to be the most widely observed mechanism in clinical cases of chemotherapy resistance. This phenomenon is often associated with the overexpression of certain ATP binding cassette transporters including P-glycoprotein (Pgp, coded by the MDR1 gene), which is an ATP dependent active efflux pump that is able to extrude a large variety of chemotherapeutic drugs from the cells [8, 9].

The conformation sensitive UIC2 mouse monoclonal antibody inhibits Pgp mediated substrate transport. However, this inhibition is usually partial and its extent is variable, since UIC2 binds only to 10–40% of all Pgp molecules present in the cell membrane [10, 11]. It has been shown earlier in* in vitro* and* ex vivo* experiments that the combined administration of UIC2 antibody and certain substrates or modulators used at low concentrations increases the antibody binding, leading to a near complete Pgp inhibition, thus providing a novel, specific, and effective way of blocking Pgp function [11, 12]. Krasznai et al. [12] also demonstrated that the combined treatment with UIC2 antibody and Pgp modulators effectively blocked the function of Pgp in ovarian carcinoma cells* in vitro* and the effect could be followed by using tumor diagnostic tracers, ^99m^Tc-MIBI and ^18^FDG.

Ovarian cancer is the second most common cancer of the female genital tract but accounts for over half of all deaths related to gynecologic neoplasms [13]. Anthracycline antibiotics have been used for more than 40 years in the treatment of gynecologic tumors in the first line in monotherapy or in combination with other drugs [14, 15].

In this paper using an animal model we demonstrate that the miniPET technique combined with tumor diagnostic radiopharmaceuticals is suitable for the detection of either Pgp expressing or Pgp nonexpressing tumors and it can be applied for* in vivo* monitoring of the effect of tumor therapy.

## 2. Materials and Methods

### 2.1. Cell Lines

Drug sensitive (Pgp^+^) cell lines and their nonsensitive (Pgp^−^) counterparts were used in the experiments. Human epidermoid (cervix) carcinoma cell lines—KB-V-1 (Pgp^+^), KB-3-1 (Pgp^−^)—and human ovarian carcinoma cell lines—A2780AD (Pgp^+^), A2780 (Pgp^−^)—were grown as monolayer cultures at 37°C in a 95% humidified air, 5% CO_2_ atmosphere. The cell lines were maintained in 75 cm^2^ flasks in Dulbecco's modified Eagle's medium (DMEM) containing 4.5 g/L glucose and supplemented with 10% heat-inactivated fetal bovine serum (FBS), 2 mM L-glutamine, and 25 *μ*M/mL gentamicin. The KB-V-1 cells were cultured in the presence of 180 nM vinblastine and the A2780AD cells were cultured with 2 *μ*M doxorubicin until use. The viability of the cells used in our experiments was always higher than 90%, as assessed by the trypan blue exclusion test.

### 2.2. Laboratory Animals

Twenty-four (10- to 12-week-old) pathogen-free B-17 severe combined immunodeficiency (SCID) female mice were used in this study. Animals were housed under pathogen-free conditions in air conditioned rooms at a temperature of 26 ± 2°C, with 50 ± 10% humidity and artificial lighting with a circadian cycle of 12 h. The diet and drinking water (sterilized by autoclaving) were available ad libitum to all the animals. The* Principles of Laboratory Animal Care* (National Institute of Health) were strictly followed, and the experimental protocol was approved by the Laboratory Animal Care and Use Committee of the University of Debrecen.

### 2.3. Animal Model and Study Design

SCID mice were injected subcutaneously (s.c.) with KB-3-1 (1.5 × 10^6^ cells in 150 *μ*L PBS) cells on the left and KB-V-1 (3 × 10^6^ cells in 150 *μ*L PBS) cells on the right side. Other groups of SCID mice were injected s.c. with A2780 (3 × 10^6^ cells in 150 *μ*L PBS) cells on the left and A2780AD (4.5 × 10^6^ cells in 150 *μ*L PBS) cells on the right side. On each side the animals received two injections of the same cell line, one in the shoulder and one in the thigh to double the tumor numbers per animal in order to limit the number of animals and to maximize the number of tumors imaged. Preliminary experiments showed that, in contrast to the Pgp^−^ cells, the tumor formation from Pgp^+^ cells has a slower kinetics. This difference was equalized by the injection of different cell numbers. Four days after the injection mice were treated with doxorubicin (DOX) (5 mg/kg) combined with UIC2 monoclonal antibody (5 mg/kg) and cyclosporine A (CSA) (10 mg/kg). Tumor growth was assessed by caliper measurements every two days by the same experienced researcher. The tumor size was calculated using the following formula: (largest diameter × smallest diameter^2^)/2 [16].

### 2.4. Radiotracers

The glucose analog (^18^FDG) was synthesized and labeled with the positron-decaying isotope ^18^F according to Hamacher et al. [17]. The radiosynthesis of the thymidine analog (^18^F-FLT) was performed according to the published method by Grierson and Shields [18]. ^11^C-Methionine was synthesized as described by Mitterhauser et al. [19]. The ^18^F-labeled nitroimidazole compound fluoroazomycin arabinoside (^18^FAZA) was performed according to the published method by Piert et al. [7].

### 2.5. Small Animal PET Imaging Using Radiopharmaceuticals

After the implantation ^18^FDG, ^11^C-methionine, and ^18^F-FLT scans were repeated at different time points. Prior to PET, mice were fasted overnight. On the day of PET imaging mice were prewarmed to a body temperature of 37°C and this temperature was maintained throughout the uptake and scanning period to minimize the brown fat visualization. Mice were injected via the tail vein with 5.0 ± 0.2 MBq of ^18^FDG, 8.1 ± 0.6 MBq of ^11^C-methionine, 4.5 ± 0.2 MBq of ^18^F-FLT, or 5.5 ± 0.5 MBq of ^18^FAZA. 20 min after ^11^C-methionine or 40 min after ^18^FDG or ^18^F-FLT or 120 min after ^18^FAZA tracer injection animals were anaesthetized by 3% isoflurane with a dedicated small animal anesthesia device. 20 min static single-frame PET scans were acquired using a small animal PET scanner (miniPET-II, Department of Nuclear Medicine, Debrecen) to visualize the tumors. On the same animal the ^11^C-methionine and ^18^FDG and ^18^F-FLT scans were made within 4 days.

The miniPET-II scanner consists of 12 detector modules including LYSO scintillator crystal blocks and position sensitive PMTs [20]. Each crystal block comprises 35 × 35 pins of 1.27 × 1.27 × 12 mm size. Detector signals are processed by FPGA based digital signal processing boards with embedded Linux operating system. Data collection and image reconstruction are performed using a data acquisition module with Ethernet communication facility and a computer cluster of commercial PCs. Scanner normalization and random correction were applied on the data and the images were reconstructed with the standard EM iterative algorithm. The voxel size was 0.5 × 0.5 × 0.5 mm and the spatial resolution varies between 1.4 and 2.1 mm from central to 25 mm radial distances [20]. The system sensitivity is 11.4%.

### 2.6. PET Data Analysis

Radiotracer uptake was expressed in terms of standardized uptake values (SUVs) and tumor to muscle (T/M) ratios. Ellipsoidal 3-dimensional volume of interest (VOI) was manually drawn around the edge of the tumor xenografts activity by visual inspection using BrainCad software (http:/www.minipetct.hu/). The standardized uptake value (SUV) was calculated as follows: SUV = [VOI activity (Bq/mL)]/[injected activity (Bq)/animal weight (g)], assuming a density of 1 g/cm^3^. The T/M ratios were computed as the ratio between the mean activity in the tumor VOI and in the background (muscle) VOI.

### 2.7. Whole-Body Autoradiography

For whole-body autoradiography the implantation of tumor cells was carried out as described above. On the 16th day after epidermoid carcinoma and on the 25th day after ovarian carcinoma cell implantation tumor-bearing mice were anaesthetized and the radioligands ^18^F-FLT (4.5 ± 0.2 MBq in 150 *μ*L saline) or ^18^FDG (5.5 ± 0.2 MBq in 150 *μ*L saline) or ^18^FAZA (5.5 ± 0.5 MBq in 150 *μ*L saline) were injected via the tail vein. Animals were euthanized 60 min after the administration of ^18^FDG or ^18^FLT and 120 min after ^18^FAZA injection with 300 mg/kg pentobarbital (Nembutal). Each animal was embedded in 1% carboxymethylcellulose solution. After being frozen in liquid nitrogen, 60 *μ*m thin cryostat sections (Leica CM 3600 cryomacrotome, Nussloch, Germany) were cut in the coronal plane. Sections were exposed to phosphorimaging plates (GE Healthcare, Piscataway, NJ, USA). For anatomic correspondence true color images of the sections were also obtained by a transparency scanner (Epson Perfection 1640, EPSON Deutschland GmbH, Meerbusch, Germany). Autoradiography and transmission images were overlaid to fuse the functional and anatomical information. For phosphorimage analysis of selected sections the ImageQuant 5.0 (GE Healthcare, Piscataway, NJ, USA) image analyzing software was used.

### 2.8. Tumor Sample Preparation

16 days after epidermoid carcinoma and 25 days after ovarian carcinoma cell implantation mice were euthanized with an overdose of pentobarbital and tumors were dissected from the animals. Tumor samples were embedded in cryomatrix and snap-frozen in liquid nitrogen. Samples were stored at −80°C in an ultralow temperature freezer.

### 2.9. Immunohistochemistry

Immunohistochemistry was performed on 5 *μ*m thick frozen tumor sections. Frozen sections were dried at room temperature and fixed in precooled acetone (−20°C) for 10 min. Sections were then washed and incubated with 0.3% H_2_O_2_ in methanol for 20 min to quench endogenous peroxidase activity. After blocking the nonspecific binding with 1% BSA for 20 minutes, sections were incubated for 60 min at room temperature with mouse anti-Pgp monoclonal antibody UIC2 (10 *μ*g/mL). After two washes with PBS, anti-mouse EnVision Detection Systems DAB (Dako, Denmark) was used to visualize the primary antibodies and sections were counterstained with haematoxylin. Negative controls were obtained by omitting the primary antibody.

### 2.10. Flow Cytometric Measurements

Formaldehyde (1% in PBS) prefixed cells were centrifuged at 500 ×g for 5 min and washed twice with 1% PBS/BSA. To visualize the Pgp expression, cells (1 × 10^6^ cells/mL) were incubated with 10 *μ*g/mL of UIC2 mAb in PBS containing 1% BSA (PBS/BSA) at 37°C for 40 min. After two washes with ice-cold PBS, the cells were incubated with rabbit anti-mouse Alexa 488 secondary antibody (10 *μ*g/mL A488-GaMIgG, Invitrogen, CA) at 4°C for 30 min. Negative controls were obtained by omitting the primary antibody. A Becton-Dickinson FACScan flow cytometer (Becton-Dickinson, Mountain View, CA) was used to determine fluorescence intensities. Emission was detected through a 540 nm broadband interference filter for Alexa 488. Cytofluorimetric data were analyzed by BDIS CELLQUEST (Becton-Dickinson) or FloWin (written by Drs. M. Emri and L. Balkay, Department of Nuclear Medicine, University of Debrecen) software.

### 2.11. Data Analysis

Data are presented as mean ± SEM of at least three independent experiments. The significance was calculated by Student's *t*-test (two-tailed). The level of significance was set at *P* ≤ 0.05 unless otherwise indicated.

## 3. Results

### 3.1. Flow Cytometric Studies

Flow cytometric analyses showed a remarkably high expression of Pgp in the Pgp^+^ cells. The ratio of the relative mean fluorescence intensities of the Pgp^+^/Pgp^−^ ovarian carcinoma (A2780AD/A2780) and the epidermoid adenocarcinoma (KB-V-1/KB-3-1) cell line pairs was 12.7 ± 2.3 and 10.0 ± 1.8, respectively.

### 3.2. Histopathological Studies

**Figure 1:**
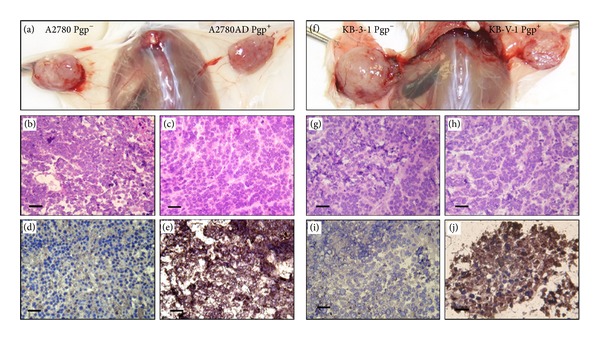
Histopathological analysis of tumor xenografts. Autopsy images show the human ovarian (a) and human epidermoid carcinoma (f) tumor xenografts 25 and 16 days after tumor cell implantation, respectively. Microscopic images of H&E staining ((b) A2780 Pgp^−^, (c) A2780AD Pgp^+^; (g) KB-3-1 Pgp^−^, (h) KB-V-1 Pgp^+^) and UIC2 mAb-DAB immunostaining ((d) A2780 Pgp^−^, (e) A2780AD Pgp^+^; (i) KB-3-1 Pgp^−^, (j) KB-V-1 Pgp^+^) of xenograft tumor sections. Bar: 50 *μ*m; magnification ×200.

SCID mice were injected in their left and right sides with Pgp^−^ (A2780, KB-3-1) and Pgp^+^ (A2780AD, KB-V-1) cells, respectively. No morphological differences were seen between the congruous Pgp^−^ and Pgp^+^ tumors upon histopathological analysis of harvested tumors by haematoxylin-eosin (H&E) staining (Figures 1(b), 1(c), 1(g), and 1(h)). Tumor cells implanted into the SCID mice retained their Pgp^+^ or Pgp^−^ phenotype as proved by immunostaining of Pgp. Immunoperoxidase detection showed strong positive staining in Pgp^+^ tumor cell membranes (Figures 1(e) and 1(j)); in contrast, no staining was visible in Pgp^−^ tumors (Figures 1(d) and 1(i)).

### 3.3. Impact of Combined Treatment on Tumor Volume

**Figure 2:**
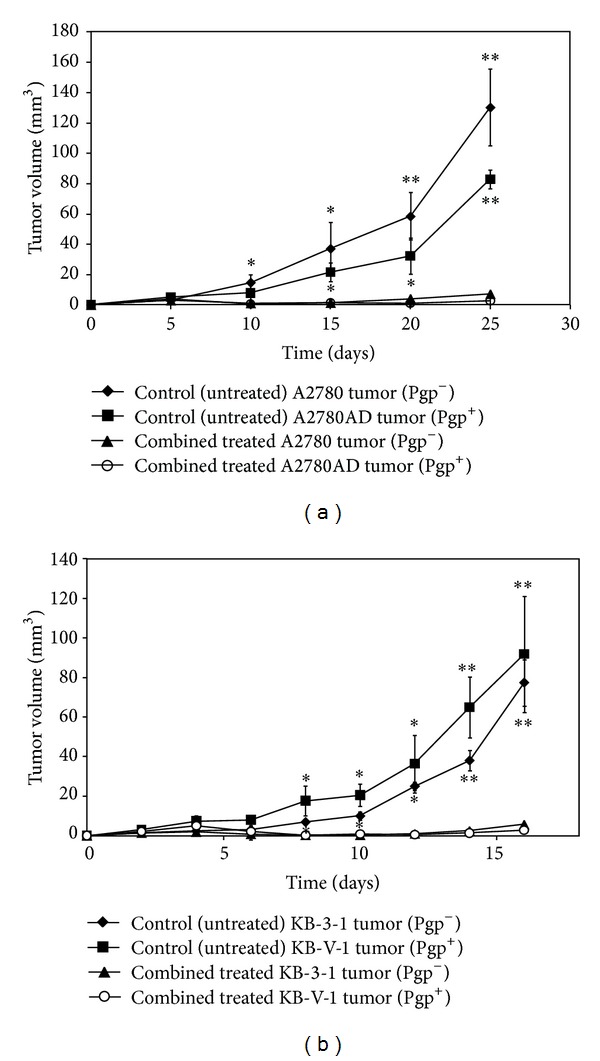
Impact of combined treatment on tumor growth. Pgp^+^ and Pgp^−^ treated tumors' volumes were compared to Pgp^+^ and Pgp^−^ control (untreated) tumors in tumor-bearing animals. Treatments began 4 days after tumor cell inoculations. (a) Impact of combined treatment on A2780AD Pgp^+^ and A2780 Pgp^−^ (6 mice, 24 tumors). (b) Impact of combined treatment on KB-V-1 Pgp^+^ and KB-3-1 Pgp^−^ tumors (6 mice, 24 tumors). Statistically significant changes in tumor volume compared to the tumor volume of untreated control are indicated (*P ≤ 0.05, **P ≤ 0.01).

Four days after the s.c. injection of tumor cells mice were treated with a combination of UIC2 monoclonal antibody, DOX, and CSA. Other groups of animals (tumor-bearing control (untreated)) were injected with PBS. The growth rate of the ovarian carcinoma tumors (A2780 Pgp^−^ and A2780AD Pgp^+^) was slower (doubling time: 4 days) than that of the epidermoid carcinoma tumors (KB-3-1 Pgp^−^ and KB-V-1 Pgp^+^; doubling time: 3 days). The growth rates of the treated tumors were compared to that of the untreated tumors in both the cases of the Pgp^+^ and the Pgp^−^ ones. In contrast to the control (untreated) tumors, where exponential growth was observed, the combined treatment inhibited the tumor growth (Figure 2). In case of the human ovarian carcinoma xenografts, from day 10 significant differences (on day 10: *P* ≤ 0.05, on day 25: *P* ≤ 0.01) were observed between the treated and untreated A2780AD and A2780 tumor volumes. These results were similar to the experiments with the human epidermoid carcinoma xenografts, where we found significant differences in the volume (from day 8: *P* ≤ 0.05, from day 14: *P* ≤ 0.01) of the control and treated tumors (Figure 2(b)).

### 3.4. miniPET Imaging of Combined Treated and Untreated Tumor Xenografts

**Figure 3:**
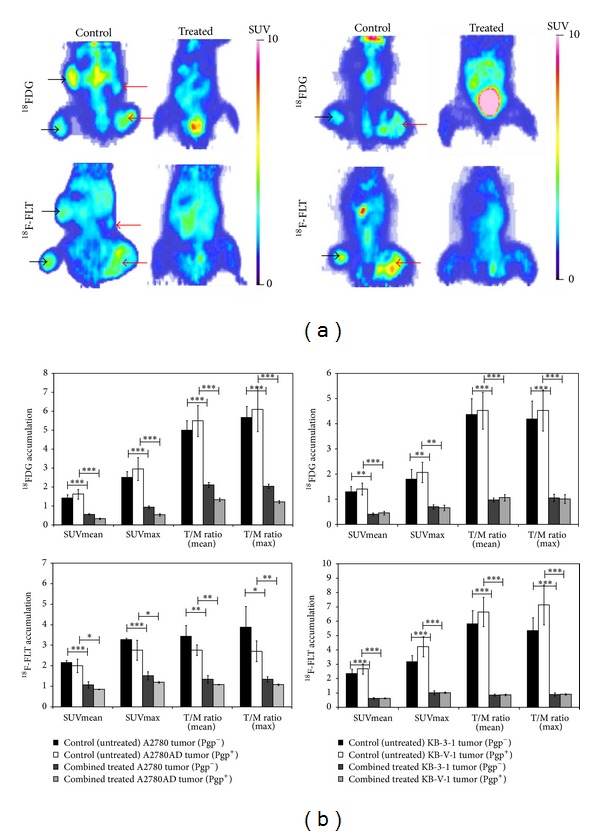
Effect of combined treatment on the ^18^FDG and ^18^F-FLT uptake of Pgp positive and negative human ovarian (left side) and epidermoid carcinoma (right side) tumors. (a) ^18^FDG and ^18^F-FLT miniPET images of control and combined treated Pgp^−^ (left side, black arrows) and Pgp^+^(right side, red arrows) tumor-bearing mice (coronal sections). No tumors can be visualized in the treated animals. (b) The SUVmean and SUVmax and the mean and maximum T/M ratios are displayed (bars represent mean ± SEM). Statistically significant differences in comparison to combined treated tumors are indicated (*P ≤ 0.05, **P ≤ 0.01, and ***P ≤ 0.001).

For* in vivo* visualization of the effect of the combined treatment on tumors ^18^FDG and ^18^F-FLT miniPET scans were performed at different time points and SUVmean, SUVmax, T/Mmean, and T/Mmax ratios were calculated (Figure 3). Fifteen to twenty days after A2780 Pgp^−^ and A2780AD Pgp^+^ cell implantation control (untreated) and combined treated tumor-bearing mice (6 mice, 24 tumors) received ^18^FDG and ^18^F-FLT. Control tumors demonstrated high ^18^FDG and ^18^F-FLT uptake (Figure 3(a), left). Quantitative image analysis showed significant differences (*P* < 0.001) between the ^18^FDG avidity of treated Pgp^+^ (SUVmean = 0.33 ± 0.03 and SUVmax = 0.54 ± 0.06) and untreated Pgp^+^ (SUVmean = 1.62 ± 0.25 and SUVmax = 2.96 ± 0.6) tumors and also between the treated Pgp^−^ (SUVmean = 0.56 ± 0.03 and SUVmax = 0.94 ± 0.07) and untreated Pgp^−^ (SUVmean = 1.43 ± 0.17 and SUVmax = 2.51 ± 0.3) tumors. By taking the T/M ratios, the differences between the treated and untreated tumors were also significant (*P* < 0.001). The uptake of ^18^F-FLT was significantly increased at the control tumors compared with that at the combined treated tumors (Figure 3(b), left). The ^18^F-FLT miniPET imaging and SUV values showed the efficiency of the combined treatment.

The biodistribution of ^18^FDG and ^18^F-FLT on days 10–15 following tumor inoculation in epidermoid carcinoma bearing mice is shown in Figure 3(a). The results of ^18^FDG and ^18^F-FLT miniPET scans showed significant accumulation in the untreated control tumors (Figure 3(a), right), in contrast to the combined treated KB-V-1 Pgp^+^ and KB-3-1 Pgp^−^ tumors.

Overall, in the treated animals the T/M ratios showed no difference in the ^18^FDG and ^18^F-FLT uptake between the place of inoculation and its muscle environment, proving the lack of tumor cells. No significant differences were observed with these two radiopharmaceuticals in the SUV values between Pgp^+^ and Pgp^−^ tumors.

**Figure 4:**
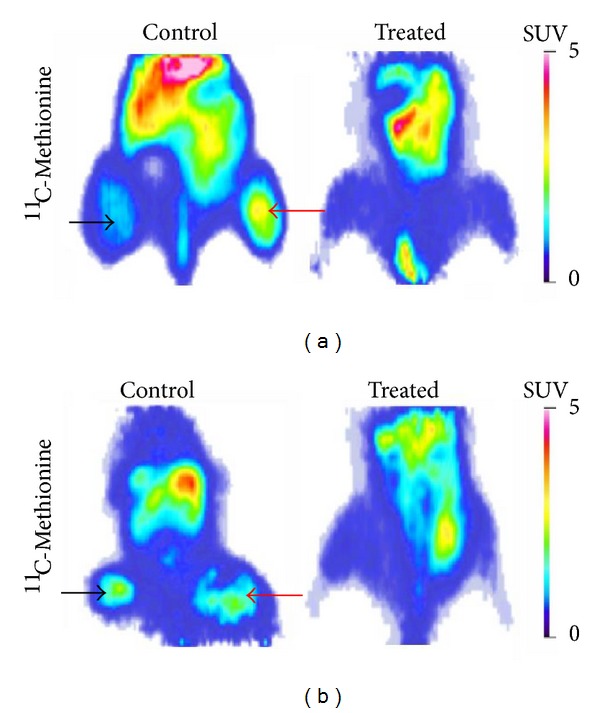
Effect of combined treatment on the ^11^C-methionine uptake of Pgp positive and negative human ovarian (a) and epidermoid carcinoma (b) tumors. ^11^C-Methionine miniPET images of control and treated Pgp^−^ (left side, black arrows) and Pgp^+^(right side, red arrows) tumor-bearing mice (coronal sections). No tumors can be visualized in the treated animals.

Fifteen to twenty days after A2780 Pgp^−^ and A2780AD Pgp^+^ cell implantation control (untreated) and treated tumor-bearing mice received ^11^C-methionine. Control tumors showed high ^11^C-methionine uptake (Figure 4(a)). Quantitative image analysis demonstrated that there were notable differences between the ^11^C-methionine avidity of the treated Pgp^+^ (SUVmean = 0.43 ± 0.02 and SUVmax = 0.83 ± 0.03) and untreated Pgp^+^ (SUVmean = 1.35 ± 0.46 and SUVmax = 2.20 ± 0.6) tumors and also between the treated Pgp^−^ (SUVmean = 0.45 ± 0.03 and SUVmax = 0.84 ± 0.06) and untreated Pgp^−^ (SUVmean = 1.35 ± 0.8 and SUVmax = 2.40 ± 1.5) tumors.

The ^11^C-methionine miniPET imaging (Figure 4(b)) and SUV values also showed the efficiency of the combined treatment in case of the epidermoid tumor xenografts. 10–15 days after KB-3-1 and KB-V-1 tumor cell implantation the quantitative image analysis showed notable differences between the ^11^C-methionine uptake of the treated Pgp^+^ (SUVmean = 0.49 ± 0.03 and SUVmax = 0.84 ± 0.02) and untreated Pgp^+^ (SUVmean = 1.52 ± 0.43 and SUVmax = 2.43 ± 0.68) tumors and also between the treated Pgp^−^ (SUVmean = 0.49 ± 0.03 and SUVmax = 0.76 ± 0.05) and untreated Pgp^−^ (SUVmean = 1.48 ± 0.46 and SUVmax = 2.55 ± 1.0) tumors.

### 3.5. Assessment of Heterogeneity in Tumor Metabolism Using Radiopharmaceuticals

**Figure 5:**
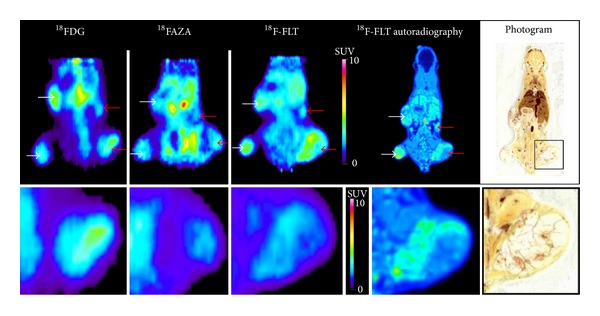
miniPET and whole-body autoradiography images from the same control tumor-bearing (ovarian carcinoma) SCID mouse (upper). Lower panels demonstrate the tumor heterogeneity using different radiopharmaceuticals on a representative Pgp^+^ tumor. White arrows: Pgp^−^ tumors; red arrows: Pgp^+^ tumors. Color bars regard to the PET images only.

The metabolic heterogeneity of control tumors was investigated on the same animal by ^18^FDG (22 days after tumor cell injection), ^18^FAZA (23 days after tumor cell injection), and ^18^F-FLT (24 days after tumor cell injection) using miniPET scanner and autoradiography techniques. Control tumors demonstrated heterogeneous ^18^F-FLT uptake, with some areas of moderate uptake surrounded by areas of intense uptake, indicating the proliferation of tumor cells. We found that the ^18^FDG positive tumor areas and the ^18^F-FLT negative areas overlapped one another. The ^18^FAZA uptake of these areas was high. After the quantitative analysis of the miniPET images the differences in the metabolic activity of the investigated tumors are shown on a representative figure (Figure 5). By taking the SUVmean values we found the following: ^18^FDG avid areas: 1.20 ± 0.27, not ^18^FDG avid areas: 0.79 ± 0.13; ^18^F-FLT avid areas: 2.67 ± 0.35, not ^18^F-FLT avid areas: 2.15 ± 0.19; ^18^FAZA avid-hypoxic-areas: 0.70 ± 0.08, not ^18^FAZA avid-not hypoxic-areas: 0.53 ± 0.07.

## 4. Discussion

Xenograft model and the miniPET technique are recently commonly used noninvasive methods in preclinical studies to detect and follow up the effect of therapy of human tumors, using PET tumor diagnostic radiopharmaceuticals, like ^18^FDG, ^18^F-FLT, ^18^FAZA, and ^11^C-methionine.

It has been demonstrated earlier by Goda et al. [11], and Krasznai et al. [12] in their* in vitro* and* ex vivo* experiments that the combined application of the Pgp specific antibody UIC2 and cyclosporine A is a successful strategy to overcome Pgp-mediated multidrug resistance.

In our experiments we used similar treatment carried out on mouse xenografts. Our aim was to test the efficacy of the combined treatment on drug sensitive and resistant human ovary and cervix cancer xenograft models by using* in vivo* medical imaging technique (miniPET) and the most commonly applied tumor diagnostic radiopharmacons (^18^FDG, ^18^F-FLT, and ^11^C-metionine) in the visualization of gynecological tumors.

The tumor cells were inoculated at four places in each animal in order to provide self-control experiment and reduce the necessary number of experimental animals. The presence of Pgp in the tumors was proved by immunohistochemistry (Figure 1).

The A2780 and A2780AD human ovarian tumors grew slower than the epidermoid adenocarcinoma tumors (KB-3-1, KB-V-1) (Figure 2). The treatment of the tumors started four days after the inoculation using a dose of 5 mg/kg doxorubicin. The size of the tumors was small (2–5 mm^3^) but visually observable. Lee et al. [21] performed efficacy studies using BALB/c mice bearing C-26 colon carcinoma tumors. In their study they applied dendrimer-DOX in which the DOX was attached by means of a stable carbamate bond in an equivalent of 20 mg/kg doxorubicin in a single dose 8 days after tumor implantation, which caused complete tumor regression and 100% survival. Kratz et al. [22] published similar results: they observed in both male and female mice an LD50 of doxorubicin 12 mg/kg. Graf et al. [23] have shown that tumor growth was inhibited by a single dose of doxorubicin ranging from 25 *μ*g to 200 *μ*g in xenograft lymphoma tumors. Kim et al. [24] treated the human ovarian A2780/DOX carcinoma xenografts with 10 mg/kg DOX three times at 3-day intervals without any effect of the MDR resistant tumor growth.

In our work we diagnosed the presence and followed the growth of multidrug sensitive and resistant tumors as well as the efficacy of the combined treatment with three tumor diagnostic tracers. The results show that the ^18^FDG (a glucose metabolic tracer), ^11^C-methionine (tracer for amino acid transport and protein synthesis), and the ^18^F-FLT (a proliferation tracer) can be effectively used for monitoring both the Pgp^+^ and the Pgp^−^ tumors. The accumulation of the tracers could be followed by definite SUV values although the ^18^F-FLT SUV values were higher than that of the ^18^FDG (Figure 3) Ong et al. [25] called attention to the fact that the PET tracer accumulation can be different in* in vitro* experiments with tumor cells and in xenografts made from the same cells* in vivo*; therefore the pharmacon uptake should be tested. Xenografts* in vivo* can be less sensitive to PET tumor diagnostic tracers than cell lines* in vitro*. In our experiments in both the A2780 and the A2780AD, as well as the KB-3-1 and KB-V-1 Pgp^+^ and Pgp^−^ cell line pairs, just as the xenografts made with these cell lines, all three tumor diagnostic tracers (^18^FDG, ^18^F-FLT, and ^11^C-methionine) could be well used. Jensen et al. [5] used ovarian cancer (A2780) xenografts in nude mice and they—similarly to our results—measured higher ^18^F-FLT than ^18^FDG uptake. On the other hand they measured approximately 50% lower SUVmean and SUVmax values compared to our results. The differences can be explained by the different experimental protocols and the biological differences between the nude and SCID mice. Ebenhan et al. [26] used KB-3-1 cervix carcinoma xenograft model (in nude mice) and found that the xenografts showed low ^18^FDG SUV and were better visualized by ^18^F-FLT. It is in agreement with other studies reporting that for assessing the early response to anticancer treatment ^18^F-FLT was superior to ^18^FDG [5]. However, other studies found higher ^18^FDG uptake compared to ^18^F-FLT accumulation in several xenograft models [27, 28]. In our experiments both tracers showed good visualization of the Pgp^+^ and Pgp^−^ tumors and the efficacy of the treatment.

^18^FDG, the most commonly used PET radiotracer in tumor diagnostics, allows visualization of the changes in the glucose metabolic rate in tumors [3]. Since Pgp is a transport ATPase, its activity may increase the ATP demand of the cells expressing it at high level [12]. The increased energy demand of the cancer cells manifests in higher glucose metabolisms that can be measured by ^18^FDG accumulation, but a number of factors can affect the FDG uptake in tumors [1, 3, 29, 30].

The different proliferation activity of the different tumors as well as the variance in the tumor volume suffering hypoxia may result in further differences in the ^18^FDG uptake (Figure 5). Similarly to our results, several authors report heterogeneity in the ^18^FDG uptake of different tumors [31, 32]. The experimental model used in our experiments provides exactly the same extracellular conditions (free glucose concentration in the blood, injected radiotracer dose, anaesthetizing procedure, etc.) since both of the Pgp^+^ and the Pgp^−^ tumors grow in the same mice; therefore the measured differences in the accumulation of the radiopharmacon show the intrinsic characters of the tumors.

In our experiments, we initiated the treatment of the tumors four days after the inoculation of the cells, when the gynecologic tumors were still rather small. We aimed to develop a model analogous to the clinical situation, when after removing the tumor by surgery a systemic therapy is applied to hinder the development of multidrug resistant primary or metastatic tumors.

Our results show that the above described combined treatment is an effective method for the chemotherapy of both Pgp^+^ and Pgp^−^ human ovarian carcinoma and epidermoid adenocarcinoma tumors growing in mice, and the efficacy of the treatment can be followed by miniPET using ^18^FDG, ^18^F-FLT, and ^11^C-methionine radiotracers. In addition, ^18^FAZA is a suitable tracer to detect the hypoxia of the tumor in xenografts. Using multitracer miniPET ^18^FDG, ^18^F-FLT, and ^18^FAZA analyses also help to detect the metabolic heterogeneity of the tumors.

## Abbreviations

CSA: Cyclosporine A
DOX: Doxorubicin
^18^FAZA: [^18^F]Fluoroazomycin-arabinofuranoside
^18^FDG: 2-[^18^F]Fluoro-2-deoxy-D-glucose
^18^F-FLT: (3′-Deoxy-3′-[^18^F]fluorothymidine)
mAb: Monoclonal antibody
MDR: Multidrug resistance
PBS: Phosphate buffered saline
PET: Positron emission tomography
Pgp: P-Glycoprotein
Pgp^+^: P-Glycoprotein positive
Pgp^−^: P-Glycoprotein negative.
